# Prevalence and risk factors for stillbirths in India: secondary analysis of ICMR-Stillbirth Pooled India Cohort (ICMR-SPIC)

**DOI:** 10.1016/j.lansea.2026.100841

**Published:** 2026-08-22

**Authors:** ICMR-SPIC Group, Pratibha Dwarkanath, John Michael Raj, Anura V. Kurpad, Tinku Thomas, Ramachandran Thiruvengadam, Ayushi, Rahul Jain, Nitya Wadhwa, Shinjini Bhatnagar, Kalpana Basany, Shailendra Dandge, Catherine L. Haggerty, P.S. Reddy, Giridhara R. Babu, Debarati Mukherjee, Deepa R, Yamuna Ana, Manisha Nair, Saswati Sanyal Choudhury, Shivaprasad S. Goudar, Manjunath S. Somannavar, Sangappa M. Dhaded, Avinash Kavi, Deepthy M. Sadanandan, Archana Patel, Kunal Kurhe, Prabir Kumar Das, Vaishali Khedikar, Alka Dafare, Chittaranjan S. Yajnik, Urmila Deshmukh, Onkar Deshmukh, Sadhana Joshi, Girija Wagh, Sanjay Lalwani, Sanjay Gupte, Juhi Nema, Ranadip Chowdhury, Rukman Manapurath, Sunita Taneja, Neeta Dhabhai, Nita Bhandari, Reema Mukherjee, Bharati Kulkarni, Ravindra Mohan Pandey

**Keywords:** Stillbirth, India, Individual participant data (IPD), Risk factors, Population attributable fraction, Maternal health, Perinatal mortality

## Abstract

**Background:**

Despite substantial reductions in stillbirth rate (SBR) over the past two decades, India continues to bear one of the highest burden of stillbirths worldwide. The available national evidence on stillbirth is fragmented, regionally variable, and often limited in scope. We aimed to determine stillbirth burden and risk factors leveraging a pooled cohort from ten prospective studies conducted across various settings in India.

**Methods:**

We established the Indian Council of Medical Research–Stillbirth Pooled India Cohort (ICMR-SPIC), combining individual-level data from ten prospective cohort studies conducted across multiple sites of India. The pooled dataset included prospective pregnancy cohort studies conducted across India between 1994 and 2023 from rural, urban, hospital, and community settings, with participant recruitment and follow-up periods varying across individual studies. After exclusions, 227,821 pregnancies were included. Harmonised analytical datasets were constructed, and weighted SBR was estimated. Multivariable mixed-effects modified Poisson regression was used to evaluate the association between stillbirth and independent maternal and antenatal risk factors. Population attributable fractions (PAFs) were calculated for modifiable exposures.

**Findings:**

A total of 3935 stillbirths occurred among 227,821 pregnancies, yielding a pooled weighted stillbirth rate of 14.8 per 1000 births (95% CI: 13.4–16.3). Independent risk factors included maternal age (>30 years) (aIRR 1.64, 95% CI: 1.55–1.72; PAF 3.23%, 95% CI: 2.97–3.48), informal maternal education (aIRR 1.45, 95% CI: 1.32–1.58; PAF 8.86%, 95% CI: 7.07–10.66), biomass fuel use (aIRR 1.37, 95% CI: 1.21–1.57; PAF 13.95%, 95% CI: 9.11–18.79), maternal height (<145 cm) (aIRR 1.32, 95% CI: 1.21–1.44; PAF 2.47%, 95% CI: 1.81–3.13), nulliparity (aIRR 1.36, 95% CI: 1.27–1.45; PAF 13.66%, 95% CI: 11.03–16.28), and previous history of stillbirth (aIRR 2.14, 95% CI: 1.15–3.97; PAF 2.13%, 95% CI: 0.97–3.29). Among obstetric complications, hypertensive disorders of pregnancy (aIRR 4.18, 95% CI: 3.54–4.94; PAF 10.27%, 95% CI: 9.74–10.81) and antepartum haemorrhage (aIRR 12.12, 95% CI: 9.54–15.40; PAF 6.33%, 95% CI: 6.19–6.47) showed strong associations.

**Interpretation:**

Our study shows that stillbirth burden remains high and factors such as women's education, biomass fuel use, hypertensive disorders, and antepartum haemorrhage were associated with higher risk of stillbirths.

**Funding:**

Indian Council of Medical Research, India.

**Research in context**

**Evidence before this study**

We searched PubMed for studies reporting on stillbirths in India. We used a combination of MeSH terms and free-text keywords (“Stillbirth” OR stillbirth∗ OR stillborn∗ OR “Foetal Death” OR “foetal death” OR “foetal death” OR “intrauterine fetal death” OR “intrauterine fetal demise” OR IUFD) AND (India). Our search was limited to articles published between January 1, 2000, and March 31, 2025. Our search identified 516 records, of which 211 had available abstracts for review. Most studies were single-site hospital case series or state-level surveys reporting stillbirth rates that varied more than three-fold depending on the data source and setting — from under 4 per 1000 births in national civil registration to over 50 per 1000 in some tertiary-care referral hospitals — with no consistent definition of stillbirth or gestational-age cut-off used across studies. Reported risk factors spanned maternal socio-demographic disadvantage, obstetric complications such as hypertensive disorders and antepartum haemorrhage, and environmental exposures such as household biomass fuel use, but almost all of this evidence came from single states, single hospitals, or single exposures analysed in isolation, with inconsistent adjustment for confounding. No study had combined individual-level data across multiple Indian cohorts to generate a single, precisely estimated national stillbirth rate or to rank risk factors by their relative contribution to the overall burden.

**Added value of this study**

This pooled individual participant data analysis of stillbirth conducted in India, combines harmonised data from 227,821 pregnancies across ten prospective cohorts conducted in diverse geographical and health-system settings. Using a standardised analytical approach with mixed-effects models, we estimated a pooled stillbirth rate of 14.8 per 1000 births and identified several maternal and antenatal characteristics that were independently associated with stillbirth, including maternal age above 30 years, informal maternal education, biomass fuel use, maternal short stature, nulliparity, previous stillbirth, hypertensive disorders of pregnancy, and antepartum haemorrhage. The harmonised participant-level dataset enabled consistent adjustment for measured confounders and accounted for between-cohort variation. Population attributable fractions were estimated to describe the relative contribution of associated factors within the observed population and should be interpreted as measures of attributable association rather than estimates of causal or preventable effects. Because most antenatal variables were measured prospectively rather than self-reported, and cohort-level random intercepts were used to account for heterogeneity across studies, our estimates are less prone to the recall bias and single-site selection effects that limited earlier evidence.

**Implications of all the available evidence**

Our findings show that India's stillbirth burden is shaped by an interacting mix of structural disadvantage and proximate obstetric complications rather than any single dominant cause. The findings of this study align with the existing evidence on stillbirth burden and its risk factors and highlights the need for effective multipronged approaches such as early detection and management of hypertensive disorders of pregnancy and antepartum haemorrhage, promotion of clean cooking fuel use and women's education, and development of pragmatic risk-stratification tools to address stillbirth. Future studies should test whether these structural factors directly cause stillbirth, or act through the obstetric complications identified here. Such initiatives could contribute to reducing stillbirth rates and support India's progress towards achieving its single-digit stillbirth target.

## Introduction

Stillbirth is a global public health problem with low-income and middle-income countries (LMICs) disproportionately bearing high burden.^1^ For international comparison, the World Health Organisation (WHO) defines stillbirth as the birth of a baby with no signs of life at or above 28 completed weeks of gestation or with a birthweight greater than or equal to 1000 g, or body length more than or equal to 35 cm when gestational age is not available. In 2021, the global stillbirth rate (SBR) was 14.3 per 1000 births translating to an estimated annual burden of 1.9 million stillbirths.^2^ Stillbirth has profound emotional, psychological, and economic consequences for families and communities.^3^ LMICs account for 98% of global stillbirth burden and India has contributed highest absolute number of stillbirths, with an estimated 397,300 stillbirths in 2021, and remains to be a highest contributor for past two decades.^1^^,^^4^^,^^5^ India, as a signatory to the global commitments of WHO's Every Newborn Action Plan (ENAP), has achieved a 53% reduction in SBR in the last two decades (29.6 stillbirths per 1000 total births in 2000 vs 13.9 in 2019) with an average annual reduction rate of 4%. However, substantial efforts are still required to achieve the ambitious target of single digit SBR by 2030 as envisioned in the India Newborn Action Plan.^6^

Considering India's marked regional and socio-economic heterogeneity, addressing the challenge of stillbirth requires context-specific data, generalisable evidence and a comprehensive understanding of its prevalence and determinants. Yet, large-scale nationally representative epidemiological studies from India remain limited, with most research being siloed to specific regions, hospitals or subpopulations, with considerable variation in case definitions. Though national surveys provide valuable demographic and health related information it suffers from several shortcomings in estimating stillbirth burden, including their cross-sectional design, retrospective reporting, and reliance on maternal recalls rather than direct observations. Such limitations introduce bias, particularly in populations with low maternal education resulting in discrepancies in stillbirth prevalence and risk factors, affecting both validity and generalisability of evidence.^7^^,^^8^

The risk factors for stillbirth are influenced by a complex interplay of maternal, foetal, and health system-related determinants. Key obstetric conditions associated with stillbirth include preeclampsia, antepartum haemorrhage, diabetes, infections, obstructed labour, anaemia, interpregnancy interval, and previous history of stillbirth. Maternal socio-demographic factors such as ethnicity, age, education, parity, nutrition, height, weight, socio-economic status, exposure to household air pollution, smoking, and access to high-quality healthcare also play an important role.^9^, ^10^, ^11^, ^12^, ^13^, ^14^ Foetal risk factors such as growth restriction, congenital anomaly, placental pathology, and health service-related risk factors such as the number of antenatal visits, the presence of a birth attendant, and the quality of intrapartum care are also known to be associated with stillbirths.^11^^,^^13^, ^14^, ^15^ However, much of this evidence comes from high-income countries or global datasets, leaving uncertainties regarding their relative contributions to stillbirth in India considering the diverse setting.^7^^,^^16^, ^17^, ^18^, ^19^, ^20^

Lack of large-scale, representative data, and imprecise estimates of prevalence and risk factors impedes the development of national policies and evidence-based interventions for reducing stillbirths in India.^5^^,^^8^^,^^11^ To address this gap, the ICMR-SPIC consortium was established.^21^ The Indian Council of Medical Research–Stillbirth Pooled India Cohort (ICMR-SPIC) is a harmonised individual-participant dataset created by pooling data from 10 pregnancy cohort studies conducted across 17 sites in nine states of India. The pooled dataset included over 229,000 pregnancies from urban, rural, and mixed settings, with prospective follow-up from pregnancy until birth. Participating cohorts met predefined eligibility criteria and contributed towards standardised data on maternal sociodemographic characteristics, pregnancy exposures, obstetric complications, and birth outcomes, enabling pooled analyses of stillbirth prevalence and associated risk factors across diverse populations in India. In this study, we estimate the stillbirth prevalence, identify associated risk factors, and their population attributable risks leveraging ICMR-SPIC dataset.

## Methods

We conducted a secondary analysis of the ICMR-Stillbirth Pooled India Cohort (ICMR-SPIC) dataset comprising ten prospective studies utilising pregnancy cohorts from India between 1994 and 2023. A detailed description of the cohort design, harmonisation procedures, participating studies, and cohort characteristics is included in our published paper.^21^ Cohorts were identified through collaborative investigator networks and relevant publications. Studies were included if they prospectively enrolled pregnant women before delivery, followed participants until end of pregnancy, and contained core variables (e.g. maternal characteristics, gestational age, and birth outcomes) required for harmonisation and pooled analysis. The studies included a mix of observational studies and randomised controlled trials drawn from both hospital and community settings across nine Indian states, coordinated through an ICMR-supported collaborative network established in 2023 (Supplementary Figure S1). For the two intervention studies (WINGS cohort and CalPreg) included (Table 1), only the control arm was included for WINGS cohort and for the CalPreg cohort, data from both arms were included because the trial reported no statistically significant difference in stillbirth incidence between the two intervention arms. After excluding 17,433 records (7.1% of original data) with missing outcome data or with abortion recorded as an outcome, the final dataset included 227,821 pregnancies (Table 1). The contributing cohorts covered a wide range of contexts, including urban (n = 4), rural (n = 4), and mixed (n = 2) sites, and together captured considerable geographic, ethnic, and health system heterogeneity (Table 1 & Supplementary Figure S1). All data were anonymised, shared under formal data-use agreements with ICMR, and derived from studies with prior ethics approval and informed consent.

**Table 1:** Cohort characteristics, study design, fetal sample sizes and exclusions in analytical datasets.

Cohort and institute	Type and setting	Study design	Time period of study	Fetal sample size	Excluded: GA <28 weeks (n)	Excluded: GA >45 weeks or missing gestational Age/Outcome (n)	Total excluded (n)	Included in analytical dataset (N)^a^	Percentage excluded (%)
Calcium in Pregnancy–SJRI, Bengaluru	Hospital, Urban	RCT	2019–2022	11,070	404	122	526	10,544	4.8
Garbhini–THSTI, Faridabad	Hospital, Mixed	Observational	2015–2020	8050	271	777	1076	6974	13.4
LIFE Cohort–MediCiti Institute, Telangana	Community-based, Rural	Observational	2010–2018	1286	12	5	17	1269	1.3
MAASTHI–IIPH Bengaluru	Hospital, Urban	Observational	2016–2020	3289	2	7	9	3280	0.3
MaatHRI–Hospitals–North East, UP, HP, MH, Chhattisgarh	Hospital, Mixed	Observational	2018–2023	10,200	1	90	91	10,109	0.9
MNHR-B–JNMC, Belagavi	Community-based, Rural	Observational	2009–2020	1,25,180	1611	11,910	13,546	1,11,634	10.8
MNHR-N–LMRF, Nagpur	Community-based, Rural	Observational	2009–2020	84,276	1010	1034	2083	82,193	2.5
PMNS–KEM Hospital, Pune	Community-based, Rural	Observational	1994–1996	797	1	26	27	770	3.4
REVAMP Study–Bharati Vidyapeeth, Pune	Hospital, Urban	Observational	2017–2020	1761	16	0	16	1745	0.9
WINGS–SAS, New Delhi	Community-based, Urban	RCT (Control)	2017–2020	1141	1	41	42	1099	3.7
Total				**2,47,050**			**17,433**	**2,29,617**	

a Additional exclusions were made due to missing outcome data in GARBHINI (n = 28), MNHR-B (n = 25), MNHR-N (n = 25), and duplicates in MNHR-N (n = 14).

A common data dictionary and harmonisation protocol were developed to standardise variable definitions and coding across cohorts. Cohort-specific datasets were mapped to this framework through investigator consultations, with recoding, unit standardisation, and logic checks applied to core variables. Derived variables were created where required, and all data were centrally reviewed with discrepancies resolved collaboratively. Multifetal pregnancies were disaggregated and analysed as separate birth records. The data harmonisation and cleaning process for the ICMR-SPIC dataset involved a collaborative effort to standardise variables across multiple consortium partners through the development of a unified data dictionary and codebook. Initial draughts were mutually refined by consortium members to establish harmonised definitions and variable names, which each cohort then used to map, recode, and rigorously clean their specific data while documenting any necessary procedural discrepancies. To ensure structural consistency, the core statistical team organised the data such that each row represented a unique pregnancy or foetus, employing specific identifiers to link related observations for accurate statistical modelling. Following this integration, the team utilised summary statistics, visualisations, and automated scripts in STATA and R to identify and correct logical errors, outliers, and missing values, ultimately excluding only those records lacking critical birth outcome data before uploading the verified sets to a secure, shared server. Detailed harmonisation procedures are described in the cohort profile paper.^21^

The primary outcome was stillbirth, defined as foetal death at or beyond 28 weeks of gestation, consistent with WHO guidelines. Gestational age was determined by ultrasound or last menstrual period, depending on the method used in the original study. Socio-demographic exposures included maternal age (categorised as ≤30 years and >30 years), education (formal vs informal/no formal education), and rural or urban residence. Maternal risk factors included short stature (height <145 cm), underweight (BMI <18.5 kg/m^2^), overweight according to the Asian classification (BMI ≥23 kg/m^2^),^22^ overweight according to the WHO classification (BMI ≥25 kg/m^2^), passive smoking (household exposure), and type of cooking fuel, classified as clean fuels (liquefied petroleum gas [LPG], natural gas, or biogas) or biomass fuels (all other fuels). Across the ICMR-SPIC cohorts, none of the women reported active smoking during pregnancy, hence it was decided to use the variable of passive smoking in analysis. Passive smoking was recorded in 77,306 participants across multiple cohorts, with 27.8% (unweighted) and 13.5% (weighted) women reporting household exposure. Obstetric history variables included nulliparity, prior stillbirth, and short inter-pregnancy interval (<18 months, where applicable). Antenatal complications were harmonised into binary variables for hypertensive disorders of pregnancy (defined according to cohort-specific protocols, predominantly based on International Society for the Study of Hypertension in Pregnancy criteria), moderate or severe anaemia at any time point in pregnancy (Hb <9.5 g/dL), antepartum haemorrhage, reproductive tract infections (defined using cohort-specific clinical, laboratory, or self-reported indicators of maternal infection during pregnancy, and intrauterine growth restriction (IUGR), defined as an estimated foetal weight below the 10th centile or more than two standard deviations below the mean for gestational age according to INTERGROWTH-21st standards at any point during pregnancy.

### Statistical analysis

The present analysis was designed as an individual participant data (IPD) pooled analysis of prospective cohort studies to estimate associations between maternal and antenatal factors and stillbirth. All estimated incidence rate ratios (IRRs) and population attributable fractions (PAFs) derived from these models should be interpreted as adjusted measures of association rather than causal effects. Descriptive characteristics of the pooled population were summarised as proportions or means with standard deviations. To account for differential representation across cohorts, all estimates were calculated as both unweighted and weighted, with weights assigned as the inverse of the proportion each cohort contributed to the total pooled sample. Stillbirth rates were expressed per 1000 total births (live births + stillbirths), with 95% confidence intervals calculated using the binomial exact method. We conducted sensitivity analyses using random-effects meta-analysis to estimate pooled stillbirth rates across cohorts, quantifying heterogeneity with the I^2^ statistic.

Associations between potential risk factors and stillbirth were examined using generalised linear mixed-effects models (GLMMs) with a Poisson distribution, log link, and robust standard variance to overcome the convergence issue with log binomial model. A random intercept for cohort was included in all models to account for within-study clustering and a small core set of covariates considered likely confounders on the basis of subject-matter knowledge and prior literature (including maternal age, parity, type of residence, type of study, and cohort) was specified a priori and retained across models where applicable, while additional candidate variables were evaluated empirically considering data completeness and harmonisation across cohorts. In the first stage, minimally adjusted models were fitted separately for each baseline (maternal age >30 years, maternal informal education, biomass fuel use, passive smoking, maternal height <145 cm, body mass index, nulliparity, delivery-to-conception interval <18 months, previous history of stillbirth) and antenatal factors ((hypertensive disorders of pregnancy, moderate or severe anaemia at any time during pregnancy [Hb < 9.5 g/dL], thyroid disorder, reproductive tract infection, antepartum haemorrhage, intrauterine growth restriction), adjusting for type of residence (rural vs urban) and type of study (community-based vs hospital-based). In the second stage, fully adjusted models were constructed separately for baseline and antenatal factors. For baseline factors, all variables that were statistically significant (p < 0.05) in the minimally adjusted models were entered simultaneously into multivariable model, along with type of residence and type of study, to derive adjusted incidence rate ratios (IRRs) and corresponding 95% confidence intervals (CIs). For antenatal factors, each variable that was statistically significant in the minimally adjusted analyses was added individually to fully adjusted model of baseline factors to assess its independent association with stillbirth, while adjusting for statistically significant baseline factors. This modelling strategy was adopted because data on antenatal risk factors were not uniformly available across cohorts, leading to variable sample sizes across models.

For exposures that remained statistically significantly associated with stillbirth in the fully adjusted models, we estimated Population Attributable Fractions (PAFs) using Miettinen's formula. These estimates were intended to rank the relative contribution of associated risk factors within this pooled dataset and should be interpreted as attributable associations rather than definitive estimates of preventable fractions because the underlying analyses are observational and residual confounding cannot be excluded:

$$PAF=P_{e}\left(RR-1\right)/RR$$

where $P_{e}$ is the proportion of stillbirth cases exposed to the risk factor, and RR is the adjusted relative risk for that risk factor derived from the fully adjusted models. All statistical analyses were performed using STATA version 16.

### Ethics statement

Ethical approval was obtained individually by each cohort's principal investigator from their respective Institutional Ethics Committees, both for conducting their original studies and for sharing data for this pooled analysis (provided in Supplementary Table). Informed consent was waived because the study used anonymised secondary datasets with no identifiable participant information.

### Role of the funding source

The funders of this research had no role in study design, data collection, data analysis or interpretation.

## Results

The harmonised ICMR-SPIC dataset includes individual-level data on 227,821 pregnant women across 10 Indian cohorts, contributing a total of 229,617 fetal observations (Table 1). The baseline demographic, obstetric, and antenatal characteristics are summarised in Table 2. In the overall study population, the mean maternal age was 23.5 years (SD 3.3) and 77.2% had formal education (mean years of education: 7.3; SD 4.8). Mean height was 152.2 cm (SD 5.6) and BMI 20.3 kg/m^2^ (SD 3.3) at enrolment, with 30.5% being underweight by South Asian standards (defined as BMI <18.5 kg/m^2^). Household characteristics included reporting of 55.5% clean fuel use and 27.9% passive smoking exposure. Obstetric profiles showed that the mean gestational age of 38 weeks and 5 days at delivery, 47.2% of women were primiparous and 1.8% had previous history of stillbirth. Common pregnancy complications included moderate or severe anaemia (33.3%) and hypertensive disorders of pregnancy (4.6%).

**Table 2:** Baseline characteristics of the ICMR-SPIC cohort.

Factors	Overall		Livebirth		Stillbirth	
	N	Mean (SD)/n (%)	N	Mean (SD)/n (%)	N	Mean (SD)/n (%)
Maternal age (Years)^a^	2,27,020	23.5 (3.3)	2,23,093	23.5 (3.3)	3927	23.8 (3.8)
Age (>30 years)	2,27,020	12,386 (5)	2,23,093	12,062 (5)	3927	324 (8)
Maternal education (years)	2,26,555	7.3 (4.8)	2,22,640	7.3 (4.8)	3915	6.7 (5.0)
Maternal Education–Informal	2,26,555	51,637 (23)	2,22,640	50,513 (23)	3915	1124 (29)
Height (cm)^a^	2,23,632	152.2 (5.6)	2,19,801	152.2 (5.6)	3831	151.2 (5.8)
Height (<145 cm)	2,23,632	15,585 (7)	2,19,801	15,199 (7)	3831	386 (10)
BMI (kg/m^2^)^a^	2,23,081	20.3 (3.3)	2,19,262	20.3 (3.3)	3819	20.3 (3.4)
BMI South Asia	2,23,081		2,19,262		3819	
Normal (18.5 to 22.9 kg/m^2^)		117,442 (53)		115,432 (53)		2010 (53)
Underweight (<18.5 kg/m^2^)		68,022 (30)		66,825 (30)		1197 (31)
Overweight (≥23 kg/m^2^)		37,617 (17)		37,005 (17)		612 (16)
BMI WHO	2,23,081		2,19,262		3819	
Normal (18.5 to 24.9 kg/m^2^)		137,067 (61)		134,749 (61)		2318 (61)
Underweight (<18.5 kg/m^2^)		68,022 (30)		66,825 (30)		1197 (31)
Overweight (≥25 kg/m^2^)		17,992 (8)		17,688 (8)		304 (8)
Residence–rural	2,19,635	201,635 (92)	2,15,853	198,145 (92)	3782	3490 (92)
Type of cooking fuel–biomass	1,01,977	45,349 (44)	1,00,238	44,458 (44)	1739	891 (51)
Passive smoking	77,306	21,532 (28)	75,983	21,151 (28)	1323	381 (29)
Nulliparous	2,27,021	119,868 (53)	2,23,100	117,991 (53)	3921	1877 (48)
Inter-pregnancy interval (≥18 months)	56,822	44,017 (77)	55,974	43,390 (78)	848	627 (74)
Previous stillbirth	2,24,228	4057 (2)	2,20,346	3903 (2)	3882	154 (4)
Hypertensive disorders of pregnancy	2,22,850	10,099 (5)	2,19,031	9585 (4)	3819	514 (13)
Moderate or severe anaemia at any time point in pregnancy (Hb < 9.5 g/dL)	1,73,623	57,783 (33)	1,70,725	56,613 (33)	2898	1170 (40)
Thyroid disorders	23,450	1179 (5)	23,034	1164 (5)	416	15 (4)
Reproductive tract infection	7294	1468 (20)	7217	1440 (20)	77	28 (36)
Antepartum haemorrhage	2,06,152	1032 (1)	2,02,591	787 (0)	3561	245 (7)
IUGR	17,535	3622 (21)	17,206	3484 (20)	329	138 (42)
Gestational age at childbirth (weeks)^a^	2,27,313	38.7 (2.5)	2,23,381	38.8 (2.4)	3932	35.4 (4.1)

N = number of participants with available (non-missing) data for the variable (the denominator); n = number of participants with the given characteristic. BMI, body mass index; IUGR, intrauterine growth restriction).

a Mean (SD); all other summary values are n (%).

Among 227,821 pregnancies in the ICMR-SPIC cohort, 3935 resulted in stillbirth. Compared with women who had livebirths, Stillbirth group included greater proportion of women with lower educational attainment, older than 30 years, and short maternal stature (<145 cm). Pregnancy complications were more frequent among women who had stillbirth, which includes hypertensive disorders of pregnancy (13% vs 4%), moderate or severe anaemia at any time during pregnancy (40% vs 33%), antepartum haemorrhage (7% vs <1%), intrauterine growth restriction (42% vs 20%), and reproductive tract infections (36% vs 20%). Stillbirth proportion was less among nulliparous women and more among those with previous history of stillbirth. Mean gestational age at delivery was lower among women who had stillbirth than those who had livebirths (35.4 vs 38.8 weeks) (Table 2). Stillbirth rate among 227,821 pregnancies was 14.78 per 1000 births (n = 3935, 95% CI: 13.43, 16.25) (Table 3). Among the cohorts, MaatHRI had the highest rate at 22.58 per 1000 births (95% CI: 19.84, 25.69), followed closely by CALPREG at 20.04 per 1000 births (95% CI: 17.52, 22.9). In contrast, MAASTHI reported the lowest rate at 3.97 per 1000 (95% CI: 2.31, 6.83), with REVAMP also showing a relatively low rate of 6.56 per 1000 (95% CI: 3.63, 11.8). The sensitivity analysis estimated stillbirth rates across different cohorts using random-effects meta-analysis and assessed regional and temporal variations using weighted rates. The overall stillbirth rate estimated through meta-analysis was 1.5% (95% CI: 1.2–1.8) (Supplementary Table 1), which was consistent with the weighted estimates, and a similar pattern of variation across cohorts was observed. Between-cohort heterogeneity in stillbirth rates was high (I^2^ = 95.3%), consistent with the wide variation in cohort-specific estimates ranging from approximately 4 to 22 stillbirths per 1000 births.

**Table 3:** Weighted stillbirth rates per 1000 births across various included cohorts.

Study	Time period	Number of pregnancies	Number of stillbirths	Weighted stillbirth rate (95% CI)
OVERALL	2019–2022	227,821	3935	14.78 (13.43, 16.25)
CALPREG^a^	2015–2020	10,480	210	20.04 (17.52, 22.9)
GARBHINI	2010–2018	6917	137	19.81 (16.78, 23.37)
LIFE	2016–2020	1269	16	12.61 (7.73, 20.49)
MAASTHI	2018–2023	3272	13	3.97 (2.31, 6.83)
MaatHRI	2009–2020	9965	225	22.58 (19.84, 25.69)
MNHR B	2009–2020	1,10,820	1874	16.91 (16.17, 17.69)
MNHR N	1994–1996	81,556	1422	17.44 (16.56, 18.36)
PUNE	2017–2020	770	8	10.39 (5.2, 20.66)
REVAMP	2017–2020	1678	11	6.56 (3.63, 11.8)
WINGS^a^	2019–2022	1094	19	17.37 (11.1, 27.08)

a Both studies were randomised controlled trials (RCTs). Only control arm data were included from the WINGS trial, whereas data from both arms of the CalPreg trial were included.

Certain maternal socio-demographic and antenatal factors showed statistically significant association with stillbirth in both minimally adjusted and fully adjusted models (Table 4). Advanced maternal age (>30 years) was associated with a 64% increased risk (adjusted IRR 1.64, 95% CI: 1.55–1.72). Informal education and short stature were linked to 45% and 32% higher risks, respectively. Among clinical conditions, antepartum haemorrhage showed the strongest association with stillbirth (adjusted IRR 12.12, 95% CI: 9.54–15.40), followed by hypertensive disorders of pregnancy (adjusted IRR 4.18, 95% CI: 3.54–4.94). Nulliparity and previous history of stillbirth were also identified as risk factors for stillbirth. Although the crude proportion of stillbirths was lower among nulliparous than multiparous women, the adjusted risk of stillbirth was higher among nulliparous women compared with multiparous. The strengthening of the point estimate after adjustment was found to be due to negative confounding by maternal age (Table 4).

**Table 4:** Maternal and antenatal risk factors for stillbirths.

Risk factors	Minimally adjusted		Fully adjusted	
	IRR (95% C.I)	P-value	IRR (95% C.I)	P-value
Maternal characteristics				
Maternal age (>30 years)	1.60 (1.53, 1.66)	<0.001	1.64 (1.55, 1.72)	<0.001
Maternal informal education	1.53 (1.40, 1.67)	<0.001	1.45 (1.32, 1.58)	<0.001
Biomass	1.42 (1.33, 1.52)	<0.001	1.37 (1.21, 1.57)	<0.001
Passive smoking	1.02 (0.98, 1.07)	0.337		
Maternal height <145 cm	1.48 (1.37, 1.60)	<0.001	1.32 (1.21, 1.44)	<0.001
BMI South Asian				
Normal (18.5–22.9 kg/m^2^)	Reference			
Underweight (<18.5 kg/m^2^)	1.02 (0.98, 1.06)	0.352		
Overweight/obese (≥23 kg/m^2^)	0.93 (0.86, 1.00)	0.06		
BMI WHO				
Normal (18.5–24.9 kg/m^2^)	Reference			
Underweight (<18.5 kg/m^2^)	1.04 (1.00, 1.08)	0.079		
Overweight/obese (≥25 kg/m^2^)	1.03 (0.88, 1.21)	0.701		
Nulliparous	1.19 (1.06, 1.34)	0.004	1.36 (1.27, 1.45)	<0.001
Delivery to conception <18 months	1.13 (0.83, 1.53)	0.451		
Previous h/o stillbirth	2.32 (1.80, 3.00)	<0.001	2.14 (1.15, 3.97)	0.016
Antenatal characteristics				
Hypertensive disorders of pregnancy	4.42 (3.36, 5.82)	<0.001	4.18 (3.54, 4.94)	<0.001
Moderate or severe anaemia at any time point in pregnancy (Hb < 9.5 g/dL)	1.30 (1.03, 1.63)	0.026	1.18 (0.92, 1.50)	0.191
Thyroid disorder	1.28 (0.68,2.41)	0.453		
Reproductive tract infection	1.58 (0.72, 3.45)	0.254		
Antepartum haemorrhage	14.93 (13.15, 16.95)	<0.001	12.12 (9.54, 15.4)	<0.001
IUGR	3.08 (2.38, 3.98)	<0.001	1.57 (0.88, 2.80)	0.127

Minimally adjusted Model: All factors were adjusted for Type of residence (Rural/Urban) and Type of study (Hospital vs Community).

Fully Adjusted model for baseline factor: Maternal age >30 years, Maternal Informal Education, Biomass, Passive smoking, Maternal Height <145 cm and Delivery to Conception <18 months, Type of residence (Rural/Urban) and Type of study (Hospital vs Community).

Fully Adjusted model for each significant Antenatal factors: each antenatal factor adjusted with factors included in fully adjusted model for baseline factors.

Population Attributable Fractions (PAF) indicated that several maternal and antenatal factors contributed to the risk of stillbirth (Table 5). Among baseline risk factors, exposure to biomass fuel (PAF: 13.95%; 95% CI: 9.11–18.79) accounted for the largest proportions of risk, followed by nulliparity (PAF 13.66%; 95% CI: 11.03–16.28), maternal informal education (PAF: 8.86%; 95% CI: 7.07–10.66). Maternal age greater than 30 years (PAF: 3.23%; 95% CI: 2.97–3.48), short maternal stature (<145 cm) (PAF: 2.47%; 95% CI: 1.81–3.13) and previous history of stillbirth (PAF 2.13%; 95% CI: 0.97–3.29) showed smaller but notable contributions. Among antenatal factors, hypertensive disorders of pregnancy accounted for 10.27% (95% CI: 9.74–10.81) of the risk of stillbirth in the population, while antepartum haemorrhage accounted for 6.33% (95% CI: 6.19–6.47). After full adjustment, the associations of moderate or severe anaemia and IUGR with stillbirth were no longer statistically significant.

**Table 5:** Population Attributable Fractions (PAF) of risk factors associated with stillbirth.

Risk factors	Prevalence of stillbirth within exposure category (%)	Prevalence of exposure category within stillbirth (%)	PAF %	95% Confidence interval	
				Lower	Upper
Maternal characteristics					
Maternal age >30 years	2.60%	8.30%	3.23%	2.97%	3.48%
Maternal informal education	2.20%	28.70%	8.86%	7.07%	10.66%
Biomass	2.00%	51.20%	13.95%	9.11%	18.79%
Maternal height <145 cm	2.50%	10.10%	2.47%	1.81%	3.13%
Nulliparous	1.90%	52.10%	13.66%	11.03%	16.28%
Previous h/o stillbirth	3.80%	4.00%	2.13%	0.97%	3.29%
Antenatal characteristics					
Hypertensive disorders of pregnancy	5.09%	13.46%	10.27%	9.74%	10.81%
Antepartum haemorrhage	23.70%	6.90%	6.33%	6.19%	6.47%

## Discussion

In this secondary analysis of 227,821 pregnancies from ten Indian cohorts, we estimated a pooled stillbirth rate of 14.8 per 1000 births and identified several socio-demographic, maternal, and antenatal risk factors to be associated with stillbirth. The use of harmonised participant-level data enabled more precise estimation of associations across diverse populations and settings. In the exploratory risk factor analysis, informal education, exposure to biomass fuels, short stature, nulliparity, and previous stillbirth history were found to be associated with a higher risk of stillbirth. Obstetric factors like hypertensive disorders of pregnancy and antepartum haemorrhage showed strong association with stillbirth. Among the risk factors analysed, biomass fuel exposure, nulliparity, maternal education, and hypertensive disorders showed the highest Population Attributable Fractions, indicating that these factors accounted for a larger proportion of the observed stillbirth burden in this pooled dataset. These PAF estimates should be interpreted as measures of attributable associations that could help to identify and prioritise potential risk factors for further investigation, rather than estimates of the proportion of stillbirths that would necessarily be prevented through intervention.

The pooled stillbirth rate observed in this study is consistent with recent national estimates and previous multicentre studies from India.^23^ Moreover, the identified risk factors such as advanced maternal age (>30 years), low maternal education, short stature, and previous history of stillbirth align with findings from global reviews^9^ and other Indian studies.^24^ Our findings are broadly consistent with previous evidence identifying hypertensive disorders of pregnancy and antepartum haemorrhage as important contributors to stillbirth.^25^, ^26^, ^27^ However, evidence on parity association with stillbirth is less consistent.^28^^,^^29^ The increased risk observed in nulliparous women of our study, after adjusting for confounding and cohort effects, suggests the need for robust studies exploring potential causal links and pathways between primigravida and a higher risk of stillbirths in future research. Moderate or severe anaemia (haemoglobin <9.5 g/dL) was associated with stillbirth risk in minimally adjusted model while it did not retain association after full multivariable adjustment for all factors, which may reflect improved programmatic screening/treatment, residual measurement differences across cohorts, or residual confounding by socio-economic factors described in earlier literature. The attenuation of associations for moderate or severe anaemia and IUGR after adjustment may also reflect overlapping pathways with other antenatal complications, residual confounding, and differences in variable completeness and harmonisation across cohorts. Considering the high prevalence of anaemia among women who had stillbirth in our dataset, we have conducted a separate in-depth analysis of its effect on stillbirth across various categories of anaemia severity and trimester, which is presented as a separate paper.

The observed associations between the identified risk factors and stillbirth are biologically plausible and align with established biological pathways and population-level epidemiology in South Asian settings.^24^ Hypertensive disorders of pregnancy and antepartum haemorrhage likely reflect shared placental pathophysiology, including placental malperfusion, and acute foeto-maternal compromise leading to hypoxia and foetal demise.^30^ Maternal short stature (<145 cm) and nulliparity are consistent with foetopelvic disproportion, dysfunctional labour, and a higher propensity for intrapartum complications, especially in contexts where timely obstetric care may be constrained. The association of advanced maternal age (>30 years) aligns with age-related vascular dysfunction, metabolic co-morbidity, oocyte and placental insufficiency, and a potentially higher prevalence of chronic hypertension and diabetes in older age groups, cumulatively reducing placental reserve.^31^

The observed association of informal education and household biomass fuel exposure with stillbirth risk likely reflect broader structural and socio-economic determinants, including poverty, living conditions, healthcare access, nutrition, and environmental disadvantage. Household biomass fuel exposure may also act through plausible biological pathways related to combustion derived particulate matter, carbon monoxide which can cross the placental barrier and induce systemic oxidative stress and inflammation, carboxyhaemoglobin-mediated foetal hypoxia, and impaired placental vascular development.^32^^,^^33^ Air pollution which is not analysed in this study could also potentially augment stillbirth risk by acting on maternal respiratory health, including pulmonary inflammation and alveolar injury, which can contribute to systemic inflammatory and vascular responses.^34^ These preliminary studies highlight a complex interplay between proximal clinical complications and upstream socioeconomic and environmental risks in the Indian population. There is a need to generate robust evidence linking biological pathways for air pollution exposure and stillbirth. Future studies should distinguish between the direct effects of air pollution and the influence of the social and environmental conditions that often accompany higher levels of air pollution.

Regional experience from South Asia, where countries such as Sri Lanka^35^ and Bhutan^36^ have achieved comparatively low stillbirth rates, offer valuable lessons to emulate. Lower stillbirth rates in Sri Lanka and Bhutan were achieved through strengthening primary maternal healthcare, skilled birth attendance, and perinatal death surveillance, placing them as critical levers for accelerating progress.^37^^,^^38^

This study has several strengths. It included individual participant-level analysis of stillbirths conducted in India, combining data from ten prospective cohorts across geographic regions, time periods, and care settings. The use of harmonised definitions, pre-specified analytic plans, and mixed-effects modified Poisson models with cohort-level random intercepts reduced heterogeneity, while ranking associated risk factors using a population attributable fraction approach provided a systematic framework for prioritising candidate exposures for future causal investigation. The use of prospectively collected maternal socio-demographic, anthropometric, household, and antenatal variables, many of them measured rather than self-reported, mitigates recall bias common to survey-based estimates. Triangulation of weighted estimates with random-effects meta-analytic summaries and sensitivity analyses supported the robustness of stillbirth burden, risk factors and PAF effect estimates.

Several limitations should be considered. Substantial heterogeneity was observed across cohorts; however, this is expected for a consortium spanning nearly three decades (1994–2023), across multiple states, including both community and hospital-based recruitment, with research questions that did not directly address stillbirth as the primary outcome. Although the primary analyses used a one-stage individual participant data approach with cohort-level random intercepts, residual heterogeneity may remain. We therefore base inference on the one-stage IPD model with cohort random intercepts and present the random-effects pooled rate only as a sensitivity analysis. Residual heterogeneity that is not captured by the random intercept is a limitation, and cohort-by-exposure interaction analyses are identified as a priority for future work. This study is intended to be a broad, mutually adjusted risk-factor screening analysis to identify factors with the strongest associations with stillbirth and to prioritise them for subsequent causal evaluation. Antenatal risk factors were not uniformly ascertained across cohorts, leading to variable sample sizes and potential selection differences in adjusted models; residual confounding is expected. Definitions and ascertainment procedures for certain antenatal complications (like antepartum haemorrhage) may also have varied across cohorts, healthcare settings, and study periods, potentially contributing to outcome misclassification and between-cohort heterogeneity. In particular, structural variables such as maternal education and biomass fuel use are closely linked with poverty, household environment, nutrition, and healthcare access, and therefore their observed associations with stillbirth should not be interpreted as directly causal until examined further. Some cohorts lacked uniform ultrasound dating or comprehensive placental/foetal pathology, which may introduce misclassification of gestational age, cause, or timing of foetal death. The wide temporal span (1994–2023) overlaps substantive improvements in obstetric and neonatal care, and although cohort effects were modelled, secular changes cannot be ignored. Hospital-based cohorts may over-represent high-risk pregnancies, whereas some community cohorts may not capture all stillbirths, potentially biasing rates despite weighting. Finally, important covariates e.g., quality of intrapartum care, transportation delays, ambient air pollution beyond biomass, and detailed infection aetiologies were unavailable or incompletely measured, which limited our ability to undertake a more comprehensive analysis of risk factors. The PAF estimates should be interpreted as measures of attributable association derived from adjusted observational models rather than as estimates of the proportion of stillbirths that would necessarily be prevented if an exposure were eliminated. Although harmonised confounder adjustment was applied, residual and unmeasured confounding remain possible. Future analyses using causal inference approaches, including directed acyclic graph-informed modelling, mediation analyses, and standardisation methods, may provide stronger evidence regarding causal pathways. Despite these limitations, the scale, harmonisation, and prospective nature of the ICMR-SPIC dataset provide valuable multi-site evidence on stillbirth burden and associated factors across diverse Indian settings.

In conclusion, stillbirth remains high in India. Several maternal, socio-demographic, and antenatal factors were associated with stillbirth across diverse Indian settings, with particularly strong associations observed for hypertensive disorders of pregnancy and antepartum haemorrhage. These findings align with the existing evidence on stillbirth burden and its risk factors and highlights the need for effective multipronged approaches such as early detection and management of hypertensive disorders of pregnancy and antepartum haemorrhage, promotion of clean cooking fuel use and women's education, and development of pragmatic risk-stratification tools to address stillbirth. These findings provide a basis for prioritising future research on developing risk stratification approaches, and informing public health planning aimed at reducing stillbirths in India.

## Contributors

RC, RT, RMuk designed the analyses with inputs from all members of the ICMR-SPIC consortium and the Technical Advisory Group (TAG). RMan, Ayushi, JMR were responsible for data cleaning and harmonisation, data preparation, and statistical analyses. KK, JN, JMR made the first draft of this manuscript. DM, MN, RC, RT, JMR, PD edited the manuscript. All authors contributed either to data acquisition, data verification, or interpretation of results. All authors had full access to all the data in the study, critically revised the manuscript, accept responsibility to submit for publication, and gave approval for the final version to be published. The consortium members contributed to the analysis plan, periodically reviewed the data preparation, analysis and contributed to interpretation of the results.

## Data sharing statement

Data and statistical codes will be available upon reasonable request.

## Editor note

The Lancet Group takes a neutral position with respect to territorial claims in published maps and institutional affiliations.

## Declaration of interests

All authors declare no competing interests.

## Appendix A Supplementary data

Supplementary Final
