# Supplementary material for: Prevalence and risk factors for stillbirths in India: secondary analysis of ICMR-Stillbirth Pooled India Cohort (ICMR-SPIC)
Source: Lancet Reg Health Southeast Asia. 2026 Aug 22;52:100841. doi: 10.1016/j.lansea.2026.100841 (PMC13711064; doi:10.1016/j.lansea.2026.100841)
Supplement: Supplementary file 1 [file mmc1.docx]

**Supplement to Prevalence and Risk Factors for Stillbirths in India: secondary analysis of ICMR-Stillbirth Pooled India Cohort (ICMR-SPIC)**

**Supplementary Figure 1: Geographical Distribution of Included Cohorts and Sample Size Contribution Across India**

**Supplementary Table 1: Random-Effects Meta-Analysis of Stillbirth Rates Across Cohorts**

| **Cohort** | **N** | **Number of stillbirths** | **Stillbirth Percentage (95% CI)** |
| --- | --- | --- | --- |
| CalPreg | 10480 | 210 | 2.00% (1.70%, 2.30%) |
| GARBH-Ini | 6917 | 137 | 2.00% (1.70%, 2.30%) |
| LIFE | 1269 | 16 | 1.30% (0.70%, 2.00%) |
| MAASTHI | 3272 | 13 | 0.40% (0.20%, 0.70%) |
| MaatHRI | 9965 | 225 | 2.30% (2.00%, 2.60%) |
| MNHR-B | 110820 | 1874 | 1.70% (1.60%, 1.80%) |
| MNHR-N | 81556 | 1422 | 1.70% (1.70%, 1.80%) |
| PMNS | 770 | 8 | 1.00% (0.40%, 2.00%) |
| REVAMP | 1678 | 11 | 0.70% (0.30%, 1.20%) |
| WINGS | 1094 | 19 | 1.70% (1.00%, 2.70%) |
| **Overall** | 227821 | 3935 | 1.50% (1.20%, 1.80%) |

**Supplemental Figure 2. Forest plot of stillbirth rate in various cohorts included in ICMR-SPIC**

*The red dotted line indicates the weighted SBR in ICMR-SPIC*

**Supplementary Table 2: Stillbirth Rate across different time periods**

| **Period** | **Number of pregnancies** | **Number of stillbirths** | **Weighted Stillbirth Rate (95% CI)** |
| --- | --- | --- | --- |
| **≤2010** | 27,048 | 565 | 1·30(1·09,1·54) |
| **2011-15** | 1,19,100 | 2082 | 1·52(1·05,2·18) |
| **2016-20** | 72,260 | 1079 | 0·71(0·46,1·10) |
| **2021-23** | 9,413 | 209 | 1·96(1·84,2·09) |

**Supplementary Figure 2: Comparison of minimally adjusted associations between maternal risk factors and stillbirth in the MNHR cohort and other cohorts.**

**Supplementary Table 3: ICMR-SPIC Consortium and Author Names**

| **Name of Cohort or Institute** | **Consortium member(s)** | **Affiliation(s)** |
| --- | --- | --- |
| Calcium in Pregnancy  (CalPreg) | Dr Pratibha Dwarkanath, PhD | Professor, Division of Nutrition, St. John's Research Institute, Bangalore |
|  | Dr John Michael Raj, MSc | Lecturer, Department of Biostatistics, St. John's Medical College, Bangalore |
|  | Dr Anura V Kurpad, PhD | Professor, Department of Physiology, St. John's Medical College, Bangalore |
|  | Dr.Tinku Thomas, PhD | Prof and Head, Department of Biostatistics, St. John's Medical College, Bangalore |
| Interdisciplinary Group for Advanced Research on Birth Outcomes - DBT India Initiative  (GARBH-Ini) | Dr Ramachandran Thiruvengadam, PhD | Associate Professor, Translational Health Science and Technology Institute, Faridabad, India |
|  | Ms Ayushi, MSc | Biostatistician, Translational Health Science and Technology Institute, Faridabad, India |
|  | Dr Rahul Jain, MBBS | Project Research Scientist-I, Translational Health Science and Technology Institute, Faridabad, India |
|  | Dr Nitya Wadhwa, MD | Senior Professor, Translational Health Science and Technology Institute, Faridabad, India |
|  | Dr Shinjini Bhatnagar, PhD | Distinguished Professor, Translational Health Science and Technology Institute, Faridabad, India |
| Longitudinal Indian Family hEalth Study  (LiFE) | Dr Kalpana Basany, MD | Professor and Head, Department of Obstetrics and Gynaecology,MediCiti Institute of Medical Sciences SHARE INDIA, Hyderabad |
|  | Dr Shailendra Dandge, MD | Professor and Head,Department of Pharmacology,MediCiti Institute of Medical Sciences, SHARE INDIA,Hyderabad, India |
|  | Dr Catherine L Haggerty, PhD | Professor, Department of Epidemiology, University of Pittsburgh School of Public Health, USA |
|  | Dr PS Reddy, MD | Professor of Medicine, University of Pittsburgh, USA |
| Maternal Antecedents of Adiposity and Studying the Transgenerational Role of Hyperglycemia and Insulin  (MAASTHI) | Dr Giridhara R Babu, PhD | Professor, Department of Population Medicine, College of Medicine, Qatar University, Doha, Qatar. |
|  | Dr Debarati Mukherjee. PhD | Professor, PHFI-Centre for Developmental and Lifecourse Research, Public Health Foundation of India, Bengaluru, India |
|  | Dr Deepa R, PhD | Research Scientistand Senior Lecturer, PHFI-Centre for Developmental and Lifecourse Research ~~,~~ Public Health Foundation of India, Bengaluru, India |
|  | Yamuna Ana, PhD | Research Associate, Indian Institute of Public Health-Bengaluru, Public Health Foundation of India, Bengaluru, India |
| Maternal and Perinatal Health Research Collaboration  (MaatHRI) | Dr Manisha Nair, PhD | Principal Investigator and Project Lead, MaatHRI project  Professor of Epidemiology & Global Health  Nuffield Department of Population Health, University of Oxford, UK  Professor of Global Health, Department of Public Health, Global Health Section, University of Copenhagen, Denmark |
|  | Dr Saswati Sanyal Choudhury | Professor of Obstetrics and Gynaecology, Guwahati Medical College, Guwahati, Assam |
| Maternal Newborn Health Registry  (MNHR)-Belagavi | Dr Shivaprasad S Goudar, MD | Professor of Physiology, J N Medical College, and Director-Research, KLE Academy of Higher Education and Research, Belagavi, India |
|  | Dr Manjunath S Somannavar, MD | Professor of Biochemistry, J N Medical College, KLE Academy of Higher Education and Research, Belagavi, India |
|  | Dr Sangappa M Dhaded, DM | Professor and Head, Department of Neonatology, J N Medical College, KLE Academy of Higher Education and Research, Belagavi, India |
|  | Dr Avinash Kavi, MD | Associate Professor of Community Medicine, J N Medical College, KLE Academy of Higher Education and Research, Belagavi, India |
|  | Dr Deepthy M Sadanandan, PhD | Research Scientist and Biostatistician, JNMC Women's and Children's Health Research Unit, J N Medical College, KLE Academy of Higher Education and Research, Belagavi, India |
| Maternal Newborn Health Registry  (MNHR)Nagpur | Dr Archana Patel, PhD | Program Director, Lata Medical Research Foundation, Nagpur, India |
|  | Dr KunalKurhe, MPH | Coordinator, Lata Medical Research Foundation, Nagpur, India |
|  | Dr Prabir Kumar Das,MD | Country Coordinator, Lata Medical Research Foundation, Nagpur, India |
|  | Dr Vaishali Khedikar,DGO | Country Coordinator, Lata Medical Research Foundation, Nagpur, India |
|  | AlkaDafare, MSc | Statistician, Lata Medical Research Foundation, Nagpur, India |
| Pune Maternal Nutrition Study  (PMNS) | Dr Chittaranjan S Yajnik, MD | Director, Diabetes Unit, KEM Hospital and Research Centre, Pune, India |
|  | Dr Urmila Deshmukh,MD | ConsultantPediatrician and Neonatologist, KEM Hospital and Research Centre, Pune, India |
|  | Mr Onkar Deshmukh, MSc | Statistician, KEM Hospital and Research Centre, Pune, India |
| Research Exploring Various Aspects and Mechanisms in Preeclampsia  (REVAMP) | Dr Sadhana Joshi, PhD | Professor and Head, Mother and Child Health, ICMR Collaborating Centre of Excellence, Interactive Research School for Health Affairs, BharatiVidyapeeth University, Pune, India |
|  | Dr GirijaWagh,MD | Professor and Head, Department of Obstetrics and Gynaecology, Bharati Hospital and Research Centre, Pune, India |
|  | Dr Sanjay Lalwani,MD | Medical Director, Professor and Head, Dept of Paediatrics, Bharati Hospital and Research Centre, Pune, India |
|  | Dr Sanjay Gupte,MD | Director, Gupte Hospital and Research Centre, Pune, India |
|  | Dr JuhiNema, PhD | Assistant Professor, Mother and Child Health, ICMR Collaborating Centre of Excellence, Interactive Research School for Health Affairs, BharatiVidyapeeth University, Pune, India |
| Women and Infants Integrated Interventions for Growth Study  (WINGS) | Dr Ranadip Chowdhury, PhD | Deputy Director and Scientist, Society for Applied Studies, New Delhi |
|  | Dr Rukman Manapurath, PhD | Society for Applied Studies, New Delhi |
|  | Dr SunitaTaneja, PhD | Senior Scientist, Society for Applied Studies, New Delhi, India |
|  | Dr Neeta Dhabhai,MD | Senior Clinical Researcher, Society for Applied Studies, New Delhi, India |
|  | Dr Nita Bhandari, PhD | Senior Scientist and Director, Society for Applied Studies, New Delhi, India |
| ICMR | Dr Reema Mukherjee, MD | Div of Reproductive Child Health and Nutrition, Indian Council of Medical Research, New Delhi |
|  | Dr Bharati Kulkarni, PhD | National Institution of Nutrition, Indian Council of Medical Research, Hyderabad |
|  | Dr Ravindra Mohan Pandey, PhD | Indian Council of Medical Research, New Delhi |

**Supplementary Table 4: Acknowledgment for research and support staff**

| **Cohort (in alphabetical order)** | **Acknowledgement** |
| --- | --- |
| Calcium in Pregnancy  (CalPreg) | Dr.Wafaie W. Fawzi, Department of Global Health and Population, Harvard T.H. Chan School of Public Health, Boston, Massachusetts, USA (the primary recipient of the multicentric grant), for allowing the use of the India trial data for this analysis. |
| Interdisciplinary Group for Advanced Research on Birth Outcomes - DBT India Initiative  (GARBH-Ini) | Department of Biotechnology, Government of India for permitting to participate in the consortium  GARBH-Ini study team (Shinjini Bhatnagar, Nitya Wadhwa, Uma Chandra MouliNatchu, Bhabatosh Das, Pallavi S Kshetrapal, Shailaja Sopory, Ramachandran Thiruvengadam, Sumit Misra, Dharmendra Sharma, Kanika Sachdeva, Amanpreet Singh, Balakrish G Nair, Satyajit Rath, and Vineeta Bal (Translational Health Science and Technology Institute, Faridabad, Haryana, India); Alka Sharma, Sunita Sharma, Umesh Mehta, and Brahmdeep Sindhu (Gurugram Civil Hospital, Gurugram, India); Pratima Mittal, Rekha Bharti, Harish Chellani, Rani Gera, Jyotsna Suri, Pradeep Debata, and Sugandha Arya (Vardhman Mahavir Medical College &Safdarjung Hospital, New Delhi, India); Arindam Maitra (National Institute of Biomedical Genomics, Kalyani, India); Tushar K Maiti (Regional Centre for Biotechnology, Faridabad, India); Dinakar M Salunke (International Centre for Genetic Engineering and Biotechnology, New Delhi, India); Nikhil Tandon, Yashdeep Gupta, Alpesh Goyal, Smriti Hari, Aparna Sharma K, and Anubhuti Rana (All India Institute of Medical Sciences, New Delhi, India); Siddarth Ramji and Anju Garg (Maulana Azad Medical College, New Delhi, India); Ashok Khurana (The Ultrasound Lab, Defence Colony, New Delhi, India); Reva Tripathi (Sitaram Bhartia Institute of Science and Research, New Delhi, India); Rakesh Gupta and Partha P Majumder (Government of India); Himanshu Sinha and Raghunathan Rengasamy (Indian Institute of Technology Madras, Chennai, India); Vineeta Bal (Indian Institute of Science Education and Research, Pune, India); Pratima Mittal (Amrita Institute of Medical Sciences & Research Centre, Faridabad, India); Uma Chandra MouliNatchu and Harish Chellani (Center for Health Research and Development, Society for Applied Studies, New Delhi, India); and Ramachandran Thiruvengadam (Pondicherry Institute of Medical Sciences, Puducherry, India).Mr AshuSharma,Technical officer-II, Translational Health Science and Technology Institute, Faridabad, India, |
| Longitudinal Indian Family hEalth Study  (LiFE) | Mr Purushotham Reddy R, Head, Information Technology, Mr Narender Konga, Data Assistant, and Mrs Deepa V Project Manager, MediCiti Institute of Medical Sciences, SHARE INDIA, Hyderabad, India |
| Maternal Antecedents of Adiposity and Studying the Transgenerational Role of Hyperglycemia and Insulin  (MAASTHI) | Ms Suganya S, PHFI-Centre for Developmental and Lifecourse Research, Public Health Foundation of India, Bengaluru, India |
| Maternal and perinatal Health research Collaboration, India  (MaatHRI) | Dr Tuck Seng Cheng, University of Oxford; Ms Rupanjali Deka, Srimanta Sankaradeva University of Health Sciences, Guwahati, and all MaatHRI site collaborators, namely;Farzana Zahir, Carolin Solomi V, Ashok Verma, Sereesha Rao, Saswati Sanyal Choudhury, Gitanjali Deka, Pranabika Mahanta, Swapna Kakoty, Robin Medhi, Shakuntala Chhabra, Anjali Rani, Amrit Bora, Indrani Roy, Bina Minzand, Omesh Kumar Bharti. |
| Maternal Newborn Health Registry  (MNHR)-Belagavi | Dr N V Honnungar, Field Research Officer, JNMC Women’s and Children’s Health Research Unit; Dr Richard J Derman, Associate Provost Global Affairs, Director Global Health Research, Professor OBGYN, Thomas Jefferson University, PA USA; Dr. Elizabeth M McClure and Ms. Janet Moore, RTI International, Durham, NC, USA;Dr. Marion Koso-Thomas, *Eunice Kennedy Shriver*National Institute of Child Health and Human Development, Bethesda, MD, USA; Dr AshalataMallapur and Dr Umesh Ramadurg, S N Medical College, Bagalkot; Medical Officers of Department of Health and Family Welfare, Government of Karnataka: Dr B D Yalgudri, Dr S M Vijapur, Dr Shivanand S Mulakuri, Dr Vrushbendra D Hiremath, Dr RamkrishnaYadgude, Dr M J Kumbar, Dr B N Kirankumar, Dr Salim Mujawar, Dr S G Shetter, Dr S C Mastiholi, Dr UdayaKudachi, Dr Shaheed S Gadekai, Dr S M Chavadappanavar, Dr R G Patil, Dr HarshaPatil, Dr S G Kamble, Dr Prashant Gaikwad, Dr R A Makhandar, Dr S SDoddmani, Dr BapusahebPatil, Dr C S Patil, Dr SukanyaHandage, Dr R S Balikai, Dr RajashekarMudhkamath, Dr S SSiddannavar, Dr Anil S Salagure, Dr Rajendra K Kilabanur, Dr Arun V Halagatti, Dr PoornimaTallur, Dr ShridharPattar, Dr (Mrs) Kusuma Magi, Dr Vishwanath M Pattar, Dr Vikas B Parvaikar, Dr Bhagya Kshatri, Dr Ashok Kuntoji; Mr. Nagaraj Khade, Mr. Sachin Mastiholi, Mr.VinayakMhetri, Mr. Dayanand Shiroor, Mr. KadappaBeniwadi |
| Maternal Newborn Health Registry  (MNHR) - Nagpur | Dr. Patricia Hibberd, Department of Global Health, Boston University School of Public Health, Boston, MA, USA; Dr. Elizabeth M McClure and Ms. Janet Moore, RTI International, Durham, NC, USA; Dr. Marion Koso-Thomas, *Eunice Kennedy Shriver* National Institute of Child Health and Human Development, Bethesda, MD, USA; Officials, Department of Health and Family Welfare, Government of Maharashtra, India; Ms. Smita Puppalwar, Dr. Rakesh Kukde, Dr. Damodar Saliwkar, Dr. Shashank Parankar, Mr. Nitesh Meshram, Lata Medical Research Foundation, Nagpur, India. |
| Pune Maternal Nutrition Study  (PMNS) | Dr Prachi Phadke and Onkar Deshmukh, KEM Hospital and Research Centre, Pune, India |
| Research Exploring Various Aspects and Mechanisms in Preeclampsia  (REVAMP) | Karuna Randhir, Interactive Research School for Health Affairs, Bharat Vidyapeeth University |
| Women and Infants Integrated Interventions for Growth Study  (WINGS) | Kiran Bhatia, Society for Applied Studies, New Delhi, India |
| ICMR, New Delhi | Ms Subhanwita Manna |

Supplementary Table 5: Details of experts in the Technical Advisory Group (TAG) for the ICMR-SPIC consortium

| Sr. No | Member name | Institution |
| --- | --- | --- |
| 1 | Dr RM Pandey (Chairperson) | Dr A.S. Paintal Distinguished Scientist Chair, ICMR New Delhi |
| 2 | Dr Anamika Gambhir | Department of Biotechnology, Govt. of India |
| 3 | Dr Shobhna Das | Ministry of Health and Family Welfare, Govt. of India |
| 4 | Dr Rakhi Dandona | Public Health Foundation of India, New Delhi, India |
| 5 | Dr Pranay Goel | Indian Institute of Science Education and Research, Pune, India |
| 6 | Dr Rajiv Sarkar | Indian Council of Medical Research, New Delhi |
| 7 | Col (Dr) Arun Kumar Yadav | Office of Director General of Armed Forces Medical Servicesin India |
| 8 | Dr K Aparna Sharma | All India Institute of Medical Sciences, New Delhi, India |

**Ethics Statement**

Ethical approval was obtained individually by each cohort’s principal investigator from their respective Institutional Ethics Committees, both for conducting their original studies and for sharing data for this pooled analysis. All cohorts had appropriate ethics and regulatory approvals, including participant consent to address research questions on stillbirth and other adverse pregnancy outcomes. The IEC ID numbers are mentioned below as follows: Cohort (in alphabetical order); Name of the Ethics Committee; and Reference ID. (1) CalPreg cohort; Institutional Ethical Review Board, St. John's Medical College, Bangalore 560034; Ref. No. 197/2017 (14 September, 2017; original); amendments and extensions: 1 January, 2018; 24 July, 2018; 9 September, 2018; 30 April, 2020; 5 June, 2020; 12 November, 2020. (2) GARBHINI cohort; Institutional Ethics Committee, Translational Health Science and Technology Institute, Faridabad, India, and Institutional Ethics Committee, Gurugram Civil Hospital, Haryana, India; THS/1.8.1/(71), dated 26 August 2019, GHG/IEC letter, dated 3 September 2019. (3) LIFE cohort; MediCiti Ethics Committee, MediCiti Institute of Medical Sciences Campus, Ghanpur, Medchal Mandal, Telangana 501401; (1) Initial study approved by IEC vide its letters dated 26 May 2008; 7 June 2008; (2) Children follow-up for 6–7 years approved by Ethics Committee vide its letter dated 31 January 2017; (3) Children follow-up for 8–16 years approved by IEC vide its letter dated 4 October 2018. (4) MAASTHI cohort; Institutional Ethics Committee at Bangalore campus of Indian Institute of Public Health - Hyderabad; Indian Institute of Public Health - Hyderabad Technical Review Committee Institutional Ethics Committee - 091-2015, dated 13 November 2015. Renewed annually until 2020. (5) MNHR-Belagavi cohort; Institutional Ethics Committee of KLE Academy of Higher Education and Research, Belagavi; Ref. No.: MDC/IECHSR/2007-08/D-6989-7002, dated 19 January 2008. (6) MNHR Nagpur cohort; Research Ethics Review Committee, Lata Medical Research Foundation, Nagpur, India; LMRF/SGCT/RPC01/01/04/2009. (7) PMNS cohort; King Edward Memorial Hospital Research Center, Pune, Maharashtra, India; KEMHRC/LFG/Dir.Offi/Adhoc/2175-A, dated 22 March 2024. (8) REVAMP cohort; Bharati Vidyapeeth Medical College Institutional Ethical Committee; IEC/2015/37, dated 3 October 2015. (9) WINGS cohort; Ethics Review Committee of the Society for Applied Studies, New Delhi, and Vardhman Medical College and Safdarjung Hospital, New Delhi, and WHO, Geneva; SAS/ERC/LG/2017 dated 3 June 2017, IEC/SJH/VMMC/PROJECT-2017/694, ERC.0002934. (10) MaatHRI cohort (Institution, Reference number and date of approval): Gauhati Medical College & Hospital: No. MC/190/2007/Pt-I/126, dated 18 January 2018. Mahatma Gandhi Institute of Medical Sciences: No. MGIMS/IEC/OBGY/118/2017, dated 15 December 2017. Institute of Medical Sciences, Banaras Hindu University: No. Dean/2018/EC/290, dated 2 January 2018. Nazareth Hospital: Ref. NH/CMO/IEC/COMMUNICATIONS/18-01, dated 2 January 2018. Emmanuel Hospital Association, New Delhi: No. 167, dated 19 January 2018. Dr Rajendra Prasad Government Medical College (Dr RPGMC), Kangra at Tanda, Himachal Pradesh, India: No. HFW-H DRPGMC/Ethics/2020/034, dated 25 June 2020. Oxford Tropical Research Ethics Committee (OxTREC): OxTREC Ref. 7-18, dated 19 April 2018. Government of India’s Health Ministry’s Screening Committee, the Indian Council of Medical Research, New Delhi: No. 2018-0152, dated 1 May 2018.
